# A combination of HLA-DP α and β chain polymorphisms paired with a SNP in the DPB1 3’ UTR region, denoting expression levels, are associated with atopic dermatitis

**DOI:** 10.3389/fgene.2023.1004138

**Published:** 2023-01-23

**Authors:** David J. Margolis, Jamie L. Duke, Nandita Mitra, Ronald A. Berna, Ole J. Hoffstad, Jenna R. Wasserman, Amalia Dinou, Georgios Damianos, Ioanna Kotsopoulou, Nikolaos Tairis, Deborah A. Ferriola, Timothy L. Mosbruger, Tristan J. Hayeck, Albert C. Yan, Dimitri S. Monos

**Affiliations:** ^1^ Department of Biostatistics, Epidemiology and Informatics, Perelman School of Medicine, University of Pennsylvania, Philadelphia, PA, United States; ^2^ Department of Dermatology, Perelman School of Medicine, University of Pennsylvania, Philadelphia, PA, United States; ^3^ Department of Pathology and Laboratory Medicine, Children’s Hospital of Philadelphia, Philadelphia, PA, United States; ^4^ Department of Pathology and Laboratory Medicine, Perelman Schools of Medicine, University of Pennsylvania, Philadelphia, PA, United States; ^5^ Section of Dermatology, Division of General Pediatrics, Children’s Hospital of Philadelphia, Philadelphia, PA, United States

**Keywords:** atopic dermatitis, case control study, human leukocyte antigens, allergy, HLA-DP

## Abstract

**Introduction:** Components of the immune response have previously been associated with the pathophysiology of atopic dermatitis (AD), specifically the Human Leukocyte Antigen (HLA) Class II region *via* genome-wide association studies, however the exact elements have not been identified.

**Methods:** This study examines the genetic variation of HLA Class II genes using next generation sequencing (NGS) and evaluates the resultant amino acids, with particular attention on binding site residues, for associations with AD. The Genetics of AD cohort was used to evaluate HLA Class II allelic variation on 464 subjects with AD and 384 controls.

**Results:** Statistically significant associations with HLA-DP α and β alleles and specific amino acids were found, some conferring susceptibility to AD and others with a protective effect. Evaluation of polymorphic residues in DP binding pockets revealed the critical role of P1 and P6 (P1: α31M + (β84G or β84V) [protection]; α31Q + β84D [susceptibility] and P6: α11A + β11G [protection]) and were replicated with a national cohort of children consisting of 424 AD subjects. Independently, AD susceptibility-associated residues were associated with the G polymorphism of SNP rs9277534 in the 3’ UTR of the *HLA-DPB1* gene, denoting higher expression of these HLA-DP alleles, while protection-associated residues were associated with the A polymorphism, denoting lower expression.

**Discussion:** These findings lay the foundation for evaluating non-self-antigens suspected to be associated with AD as they potentially interact with particular HLA Class II subcomponents, forming a complex involved in the pathophysiology of AD. It is possible that a combination of structural HLA-DP components and levels of expression of these components contribute to AD pathophysiology.

## 1 Introduction

Atopic dermatitis (AD) is one of the most common dermatologic illnesses with a known genetic predisposition (Abuabara et al., 2019a; Abuabara et al., 2019b; Chiesa Fuxench et al., 2019; Silverberg et al., 2019). It is often hypothesized to be a disorder involving both skin barrier and immune system dysfunction, the latter of which is thought to be mediated by antigen and centered on the activation of the T_H_2 pathway (Gough and Simmonds, 2007).

The Human Leukocyte Antigen (HLA) region of chromosome 6 is frequently associated with immune mediated illnesses (Gough and Simmonds, 2007). Using next-generation sequencing technology (NGS), we recently evaluated associations between HLA Class I genetic variation and AD (Margolis et al., 2021a; Margolis et al., 2021b). HLA Class II molecules, as part of the adaptive immune response, play a major role in the presentation of antigens to CD_4_ T cells, so we extended our study to include the characterization by NGS of HLA Class II polymorphisms. Presentation of antigen through the skin occurs *via* antigen presenting cells that contain receptors formed by highly polymorphic molecules (epitopes) in HLA Class II as HLA-DR, HLA-DQ, and HLA-DP (Couture et al., 2019). These genes and the subsequent receptor epitopes from these genes create highly variable HLA Class II binding groves that interact with antigen(s) (Couture et al., 2019; Gong et al., 2020). The formed epitopes within these grooves have varying affinities for antigen(s) thereby playing an important role in antigen presentation (van Lith et al., 2010; Couture et al., 2019; Gong et al., 2020). The functioning of HLA Class II is, therefore, consistent with the hypothesized immunologic basis of AD.

In the past, large genome wide association studies (GWAS) relied on imputation protocols for HLA and evaluated HLA associations in cohorts of primarily Europeans and Asians with AD (Paternoster et al., 2011; Sun et al., 2011; Weidinger et al., 2013; Paternoster et al., 2015). These reports identified HLAs associated with AD or other allergic illnesses, such as asthma and food allergy, which are diseases commonly seen with AD. These HLA associations were most frequently in the HLA Class II region (Weidinger et al., 2010; Paternoster et al., 2011; Hirota et al., 2012; Weidinger et al., 2013; Paternoster et al., 2015). Given the critical role of HLA Class II genes in the adaptive immune response, prior identification of specific HLA polymorphisms relevant to AD, and the potential role that HLA Class II polymorphisms may play in AD susceptibility/protection, deeper exploration of HLA Class variation is warranted.

Several smaller studies using older immunogenotyping technology have explored the relationship between HLA allelic variation and AD as well as comorbid “atopic” illness like asthma, food allergies, and seasonal allergies with conflicting results (Paternoster et al., 2011; Sun et al., 2011; Weidinger et al., 2013; Margolis et al., 2015; Paternoster et al., 2015). For example, Aron et al. found a strong positive relationship between HLA Class II DR4 and DR7 alleles with atopy and asthma in a Finnish cohort (Aron et al., 1996). HLA-DRB1, -DQB1 and -DPB1 genotypes were shown to be correlated with peanut allergy in a British study (Howell et al., 1999). In a small Japanese study, HLA-DRB1 and -DQB1 alleles were associated with severe AD with high IgE levels (Saeki et al., 1995). In contrast, Affes et al. found no association between AD and HLA-B, -DR and -DQ, whereas HLA-A had a protective effect in a cohort of Tunisian patients (Affes et al., 2007). Park et al. showed an association of HLA-DRB1 with AD in Korean children with food allergy (Park et al., 2012). In a small study of African-Americans, HLA-DRB1 allelic variation was specifically associated with HLA-DR receptor binding-pocket changes that were associated with both the onset and persistence of AD; these associations were not found in Whites (Margolis et al., 2015). Madore et al. reported an association between HLA-DQB1 and peanut allergy (Madore et al., 2013).

Employing advances in sequencing technologies, we performed HLA gene targeted sequencing to fully characterize the HLA genes in an AD case-control cohort [*Genetics of Atopic Dermatitis* (GAD) cohort], where the goal of this study was to determine whether genetic variation of these genes is associated with the likelihood of susceptibility to or protection from AD. Next-generation sequencing (NGS)-based technologies is now common in clinical practice for HLA genotyping labs supporting transplant programs but is still rarely performed for large-scale disease association cohort studies. We conducted a comprehensive and detailed characterization of HLA genes (including exonic, intronic, and some 3’ UTR sequences). Previously, we utilized this cohort to study the Class I HLA genes and found one allele associated with AD, B*53:01, and four alleles (A*01:01, A*02:01, B*07:02 and C*07:02) and six HLA protein residues (HLA-A 9F, HLA-A 97I, HLA-A 152V, HLA-A 156R, HLA-B 163E, and HLA-C 116S) that were associated with protection from AD (Margolis et al., 2021b). Our current study undertook a different approach to study the HLA Class II genes, taking into account the nature of the Class II genes to form dimers of the alpha and beta genes on the cell surface to present peptides. First, we evaluated allelic variation in the GAD cohort for the HLA Class II genes HLA-DRB1, DQA1, DQB1, DPA1, and DPB1. Based on the allelic variation, we assessed the differential frequency of specific amino acid residues comprising the binding pockets of the -DR molecules (Zerva et al., 1996; Raychaudhuri et al., 2012; van Deutekom and Keşmir, 2015). Next, we evaluated the differential frequency of HLA Class II A1-B1 haplotypes formed for the DQ and DP dimers and the pocket residues, critical for peptide binding, within each of the DQ or DP dimers. HLA-DRA is not polymorphic so it is not necessary to characterize the HLA-DRA1/-DRB1 dimers (Matern et al., 2020). Additionally, we also assessed the polymorphisms of the other, non-antigen recognition domains, DQ or DP α2, DQ or DP β2 domains, the transmembrane and cytoplasmic regions, and a 3’ UTR of DPB1 single nucleotide polymorphism (SNP) rs9277534, known to be associated with DP expression (Thomas et al., 2012; Schöne et al., 2018). A replication cohort, The Pediatric Eczema Elective Registry (PEER) was also HLA characterized by NGS and used to confirm the associations identified with the GAD cohort. Our set of analyses, which relied on the detailed characterization of HLA genes *via* NGS, help to further our understanding of the genetic basis of AD. Moreover, this analytic framework can potentially serve as a model for future studies of HLA polymorphisms identified through NGS in a range of diseases.

## 2 Materials and methods

### 2.1 Population

Our primary study was comprised of 849 subjects from the *Genetics of Atopic Dermatitis* (GAD) cohort that included 464 cases (AD) and 385 controls (who did not have AD) (Margolis et al., 2015; Margolis et al., 2021b; Margolis et al., 2021c). All subjects were examined by dermatologists with expertise in the diagnosis of AD from the following Dermatology practice locations: University of Pennsylvania Perelman School of Medicine, Children’s Hospital of Philadelphia, Pennsylvania State University/Hershey Medical Center, and Washington University School of Medicine in St Louis. All subjects had a history and an exam consistent with AD (cases) or no history of AD (controls). There was no age restriction for enrollment. HLA polymorphisms, as well as AD genetic variation, can be highly dependent on race, so we focused on White and Black Americans in our study (Margolis et al., 2019). All subject-related information was obtained using a standard case report form that was completed by the subject or by an investigator after subject interview and/or medical record review.

All subjects or legal guardians provided written informed consent or, if appropriate, assent approved by their appropriate Institutional Review Board.

After completion of the study, a replication cohort of 424 AD subjects, called the Pediatric Eczema Elective Registry (PEER) was HLA characterized and used to assess the findings in the GAD cohort. This registry is described in detail is previous publications and compared to the 385 GAD controls (Margolis et al., 2012).

### 2.2 HLA genotyping

DNA was collected using Oragene DNA collection kits (DNA Genotek, Ottawa Canada) as previously reported (Margolis et al., 2012). The five HLA Class II genes (DRB1, DPA1, DPB1, DQA1, and DQB1) for individuals in the GAD cohort were sequenced using targeted amplicon-based NGS with Omixon Holotype HLA™ V2 kits (Budapest, Hungary). HLA genes were amplified (Qiagen LR PCR kits, Valencia, CA) on a Veriti thermal cycler (ThermoFisher, Waltham, MA), then amplicons from each gene were pooled per sample with library preparation occurring according to the manufacturer’s protocol. The final library was sequenced on an Illumina MiSeq (San Diego, CA) using paired-end 2 × 150 V2 chemistry. Omixon Twin™ (7,000 pairs/locus, v, 2.5.1) and GenDx NGSengine^®^ (Utrecht, Netherlands, 300,000 pairs/sample) analyzed each set of Fastq files. Genotyping was conducted in the Immunogenetics Laboratory of Children’s Hospital of Philadelphia, a CLIA and ASHI accredited clinical laboratory, using clinical protocols with appropriate quality controls and standards.

### 2.3 Data analysis

The NGS sequencing included full genomic characterization of HLA-DPA1, -DQA1, -DQB1 genes and partial characterization for HLA-DPB1 and -DRB1 (exon 2 to the 3’ UTR). All results were presented at two field resolution. Protein variations were determined using the IPD-IMGT/HLA database (Robinson et al., 2020). We focused on known binding pocket residues for -DR, - DQ, and -DP as being critical for peptide binding and therefore possibly involved in disease processes.

Allelic frequencies (AF) were based on the number of chromosomes with alleles that coded for the residue variant and were estimated along with 95% confidence intervals (CI). Epitope or Residue frequencies (RF) were based on the number of chromosomes with alleles that coded for the residue variant. Frequencies were estimated separately for those with and without AD. Logistic regression was used to estimate the odds ratio (OR) of having AD, assuming an additive genetic model for the allele or residue. Additional analyses were conducted within racial subgroups. Amino acid residue analysis was restricted to polymorphic residues within the binding pocket of the HLA molecules. Binding pocket residues were identified based on published crystal structures for each HLA molecule, whereby the residues that were found to be within a 4-Å neighborhood of the presented peptide were included (Stern et al., 1994; Chicz et al., 1997; Henderson et al., 2007; Dai et al., 2010; Tollefsen et al., 2012; Kusano et al., 2014; Jiang et al., 2019; Ting et al., 2020).

The mature Class II molecules are composed of an α and a β chain, products of the respective A1 and B1 genes of each of the DQ and DP Class II genes. The α1 domain of the α chain and the β1 domain of the β chain form the binding site. Since both chains are polymorphic, at least for DQ and DP molecules, we explored the possible contributions generated upon formation of the dimer, and therefore the concept of haplotypic analysis. Haplotype analysis was conducted using the BIGDAWG package in R (version 3.6.2), which utilizes the R-routine *haplo.stats* and *haplo.em* to estimate haplotypes, to account for the eventual dimerization of particular pairs of α and β chains (Pappas et al., 2016). Significance was assessed using a chi-squared test. The results of using the BIGDAWG package was further confirmed by using an independent approach for haplotype generation. Haplotype analysis was only performed for the HLA-DPA1/-DPB1 and HLA-DQA1/-DQB1 pairs of genes. Note that the haplotype analysis refers to the A1 and B1 genes of a single isotype and does not refer to the extended DP ∼ DQ haplotype. Haplotype analysis was not performed for the HLA-DRA/DRB1 pair of genes since DRA is not polymorphic (Matern et al., 2020) and not characterized in our study. The DRB1 gene was therefore evaluated assessing all polymorphisms located on the DRβ chain and participating in the formation of the DR binding pockets. To identify relevant residues on the α/β chains of DP and DQ molecules influencing AD, we used alleles or haplotypes with significant *p*-values showing opposing directionality for their association with AD, and identified polymorphic residues, focusing on those that participate in pocket formation. The described approach has been previously used to identify residues of relevance for tuberculoid leprosy on the DR molecules (Zerva et al., 1996).

For completeness, all remaining polymorphic residues that were outside of exon 2 of each A1 and B1 genes (exons 1, 3, 4, 5, 6, depending on the particular gene), were analyzed and evaluated for association using logistic regression in R. Specifically, for the DPB1 gene, an additional polymorphism SNP rs9277534 at the 3’ UTR was inferred based on HLA-DPB1 genotyping (Schöne et al., 2018). SNP rs9277534 is previously reported to be associated with expression levels of DP molecules and was evaluated as high or low HLA-DPB1 expression using logistic regression for its contribution to protection or disease (Thomas et al., 2012).

To further explore the relationship and the possible independent effect of significant amino acid positions and residues found in different regions of the DPA1 and DPB1 gene, including the A/G polymorphism of rs9277534 SNP, we assessed linkage disequilibrium (LD) among these entities. LD was calculated in the whole GAD cohort using the LD coefficient D (D = p_AB_ – p_A_p_B_, where p_AB_ is the frequency of the amino acid residue of interest and rs9277534 SNP of interest co-occuring on the same haplotype, p_A_ = frequency of the amino acid residue of interest, p_B_ = frequency of rs9277534 SNP of interest; generally ranging from −0.25 to 0.25 but is dependent upon allele frequency). Further, the correlation coefficient, *R* ^2^, was calculated using D to account for the frequency of the alleles in the population (*R* ^2^ = D/(p_A_ (1-p_A_)p_B_ (1-p_B_) (Slatkin, 2008). The same strategy was used to evaluate the LD between two amino acid residues in different DPA1 or DPB1 regions, replacing the polymorphisms of rs9277534 SNP with a second amino acid residue of interest in the above calculations.

An additional type of analysis was undertaken involving the possible relationship of T cell epitope (TCE) groups with AD. Each TCE group is defined by a distinct combination of amino acid residues that influence the peptides bound to the HLA-DP protein and presented to T cells. Originally, and in the context of hematopoietic stem cell transplantation, the HLA-DPB1 alleles were organized into TCE groups by experimental methods by Zino et al. (Zino et al., 2007). The definitions of TCE groups was further extended to all alleles using a functional distance method by Crivello et al. (Crivello et al., 2015). The TCE classification is based on exon 2 amino acid composition and an updated TCE classification is maintained for all alleles as part of the IPD-IMGT/HLA database (Robinson et al., 2020) using the Crivello functional distance method (Crivello et al., 2015). HLA-DPB1 alleles from both the GAD and PEER cohorts were assigned the TCE group that is defined by the Crivello functional distance method available through the IPD-IMGT/HLA database. For the novel DPB1 alleles that exist in either the GAD or PEER cohort, all novelties are outside of exon 2, and were assigned a TCE group that corresponds to the known allele that shares exact exon 2 sequence with the novel allele.

The odds ratios were not adjusted for other atopic illnesses like asthma, seasonal allergies, or food allergies because these illnesses are likely on the same causal pathway as noted in studies of the atopic March (Kapoor et al., 2008; Davidson et al., 2019). Our final analysis was at the amino acid level within the binding pocket of the HLA molecules (e.g., a phenotype). We report the Bonferroni correct threshold by number of variants or residues analyzed per HLA gene. We also report the uncorrected *p*-value. All statistical analyses were conducted using Stata Version 17.0 (College Station, TX) or R (R Foundation, version 3.6.2).

## 3 Results

The GAD cohort consisted of 849 total subjects. Genotyping was available for -DRBI from 803 subjects, for -DPA1 from 783 subjects, for -DPB1 from 784 subject, for -DQA1 from 782 subjects, and for -DQB1 from 774 subjects. As previously described (Margolis et al., 2021b), in the entire GAD cohort 59.2% (*n* = 503) reported their race as White, 37.1% (*n* = 315) reported their race as Black, 56.2% (*n* = 477) were Female (*n* = 477) and 54.6% (*n* = 464) had atopic dermatitis. For the 464 individuals in the GAD cohort with AD, 50.0% (*n* = 203) were White, 43.8% (*n* = 203) were Black, 63.5% (*n* = 63.5%) were Female. For those with AD, the median age of onset of AD was 0.75 years (interquartile range (IQR): 0.25–11), however the median age when a sample was obtained for this study was 54.0 years (IQR: 38.4–63.7) for those with AD and 51.9 years (IQR: 35.0–65.9) for the controls. Overall, 45.1% (*n* = 342) of GAD had seasonal allergies and 39.3% (*n* = 290) had asthma. Within the AD sub-cohort, 64.4% had seasonal allergies (*n* = 290) and 56.4% (*n* = 254) had asthma. As expected and previously reported (Paternoster et al., 2011; Sun et al., 2011; Weidinger et al., 2013; Paternoster et al., 2015), seasonal allergies and asthma are significantly different in the case and control groups (*p* < 0.001)*.* Furthermore, the female subjects are significantly increased among those with AD as compared to the control group (*p* < 0.001) (Chiesa Fuxench et al., 2019; Silverberg et al., 2019).

Sixteen HLA-DRB1, ten HLA-DQA1, ten HLA- DQB1, four HLA-DPA1, and seven HLA-DPB1 alleles had a frequency of ≥0.05 in the full GAD cohort, the White sub-cohort, or the Black sub-cohort (Table 1, all alleles in Supplemental Table 1). Allelic frequency did vary by race and presence or absence of disease (Table 1). After statistical correction no DRB1 alleles were associated with AD. No DQA1 alleles and one DQB1 allele was significantly associated with AD after correction; DQB1*03:19 (2.45 (1.35, 4.44); *p* = 0.003). This allele is more common in Blacks than Whites (frequency <0.01). The low AF in Whites likely resulted in an unstable effect estimate for Whites (Table 1; Table 2). Three DPA1 alleles were significantly associated after correction with AD. DPA1*01:03 (0.60 (0.49, 0.73); *p* = 5.92 × 10^−07^) was associated with a decreased risk of AD and DPA1*02:01 (1.49 (1.17, 1.91); *p* = 0.0013), and DPA1*02:02 (1.78 (1.22, 2.59); *p* = 0.0028)) were significantly associated with an increased risk of AD. Finally, DPB1*04:01 (0.57 (0.46, 0.71); *p* = 7.81 × 10^–07^) was significantly associated with a decreased risk of AD. Effect estimates did vary by race, however, the 95% CI overlapped (Table 2).

**TABLE 1:** Allelic frequency based on chromosome with 95% CI for DRB1, DQA1, DQB1, DPA1, and DPB1 for variants with at least a frequency of >0.05 by case and control status and by race.

	Control		Case		White control		White case		Black control		Black case	
Allele	n	AF (95%CI)	n	AF (95%CI)	n	AF (95%CI)	n	AF (95%CI)	n	AF (95%CI)	n	AF (95%CI)
DPA1*01:03	517	0.72 (0.68,0.75)	496	0.59 (0.55,0.62)	426	0.80 (0.76,0.83)	330	0.76 (0.72,0.80)	88	0.48 (0.41,0.56)	141	0.39 (0.34,0.44)
DPA1*02:01	127	0.18 (0.15,0.21)	207	0.24 (0.22,0.28)	82	0.15 (0.12,0.19)	69	0.16 (0.13,0.20)	45	0.25 (0.19,0.32)	128	0.35 (0.30,0.40)
DPA1*02:02	40	0.06 (0.04,0.07)	84	0.10 (0.08,0.12)	11	0.02 (0.01,0.04)	21	0.05 (0.03,0.07)	28	0.15 (0.10,0.21)	51	0.14 (0.11,0.18)
DPA1*03:01	14	0.02 (0.01,0.03)	34	0.04 (0.03,0.06)	3	0.01 (0.00,0.02)	6	0.01 (0.01,0.03)	11	0.06 (0.03,0.11)	29	0.08 (0.05,0.11)
DPB1*01:01	91	0.13 (0.10,0.15)	126	0.15 (0.13,0.18)	36	0.07 (0.05,0.09)	18	0.04 (0.02,0.06)	55	0.30 (0.23,0.37)	104	0.29 (0.24,0.34)
DPB1*02:01	95	0.13 (0.11,0.16)	98	0.12 (0.10,0.14)	76	0.14 (0.11,0.17)	64	0.15 (0.12,0.18)	19	0.10 (0.06,0.16)	32	0.09 (0.06,0.12)
DPB1*03:01	66	0.09 (0.07,0.11)	72	0.09 (0.07,0.11)	56	0.10 (0.08,0.13)	48	0.11 (0.08,0.14)	10	0.05 (0.03,0.10)	20	0.06 (0.03,0.08)
DPB1*04:01	254	0.35 (0.32,0.39)	197	0.23 (0.21,0.26)	216	0.40 (0.36,0.45)	141	0.32 (0.28,0.37)	36	0.20 (0.14,0.26)	42	0.12 (0.08,0.15)
DPB1*04:02	81	0.11 (0.09,0.14)	92	0.11 (0.09,0.13)	65	0.12 (0.10,0.15)	61	0.14 (0.11,0.18)	15	0.08 (0.05,0.13)	30	0.08 (0.06,0.12)
DPB1*17:01	18	0.02 (0.01,0.04)	37	0.04 (0.03,0.06)	9	0.02 (0.01,0.03)	11	0.03 (0.01,0.04)	9	0.05 (0.02,0.09)	26	0.07 (0.05,0.10)
DPB1*18:01	10	0.01 (0.01,0.03)	13	0.02 (0.01,0.03)	0	0.00 (0.00,0.01)	0	0.00 (0.00,0.01)	10	0.05 (0.03,0.10)	13	0.04 (0.02,0.06)
DQA1*01:01	70	0.10 (0.08,0.12)	69	0.08 (0.06,0.10)	61	0.11 (0.09,0.14)	44	0.10 (0.07,0.13)	9	0.05 (0.02,0.09)	22	0.06 (0.04,0.09)
DQA1*01:02	151	0.21 (0.18,0.24)	203	0.24 (0.21,0.27)	96	0.18 (0.15,0.22)	80	0.18 (0.15,0.22)	55	0.30 (0.23,0.37)	112	0.31 (0.26,0.36)
DQA1*01:03	42	0.06 (0.04,0.08)	51	0.06 (0.05,0.08)	36	0.07 (0.05,0.09)	29	0.07 (0.04,0.09)	6	0.03 (0.01,0.07)	18	0.05 (0.03,0.08)
DQA1*01:05	18	0.02 (0.01,0.04)	28	0.03 (0.02,0.05)	7	0.01 (0.01,0.03)	8	0.02 (0.01,0.04)	10	0.05 (0.03,0.10)	19	0.05 (0.03,0.08)
DQA1*02:01	96	0.13 (0.11,0.16)	84	0.10 (0.08,0.12)	73	0.14 (0.11,0.17)	56	0.13 (0.10,0.16)	23	0.12 (0.08,0.18)	28	0.08 (0.05,0.11)
DQA1*03:01	51	0.07 (0.05,0.09)	54	0.06 (0.05,0.08)	42	0.08 (0.06,0.10)	38	0.09 (0.06,0.12)	9	0.05 (0.02,0.09)	20	0.06 (0.03,0.08)
DQA1*03:03	51	0.07 (0.05,0.09)	63	0.07 (0.06,0.09)	37	0.07 (0.05,0.09)	26	0.06 (0.04,0.09)	14	0.08 (0.04,0.12)	34	0.09 (0.07,0.13)
DQA1*04:01	37	0.05 (0.04,0.07)	46	0.05 (0.04,0.07)	19	0.04 (0.02,0.06)	15	0.03 (0.02,0.06)	18	0.10 (0.06,0.15)	29	0.08 (0.05,0.11)
DQA1*05:01	75	0.10 (0.08,0.13)	76	0.09 (0.07,0.11)	42	0.08 (0.06,0.10)	45	0.10 (0.08,0.14)	15	0.08 (0.05,0.13)	25	0.07 (0.04,0.10)
DQA1*05:05	98	0.14 (0.11,0.16)	123	0.15 (0.12,0.17)	37	0.07 (0.05,0.09)	69	0.16 (0.13,0.20)	24	0.13 (0.08,0.19)	51	0.14 (0.11,0.18)
DQB1*02:01	75	0.10 (0.08,0.13)	74	0.09 (0.07,0.11)	60	0.11 (0.09,0.14)	45	0.10 (0.08,0.14)	15	0.08 (0.05,0.13)	23	0.07 (0.04,0.10)
DQB1*02:02	76	0.11 (0.08,0.13)	86	0.10 (0.08,0.13)	49	0.09 (0.07,0.12)	44	0.10 (0.07,0.13)	27	0.15 (0.10,0.21)	42	0.12 (0.09,0.16)
DQB1*03:01	119	0.17 (0.14,0.19)	130	0.16 (0.13,0.18)	100	0.19 (0.16,0.22)	94	0.22 (0.18,0.26)	16	0.09 (0.05,0.14)	29	0.08 (0.06,0.12)
DQB1*03:02	62	0.09 (0.07,0.11)	67	0.08 (0.06,0.10)	50	0.09 (0.07,0.12)	39	0.09 (0.06,0.12)	12	0.07 (0.03,0.11)	24	0.07 (0.04,0.10)
DQB1*03:03	34	0.05 (0.03,0.07)	27	0.03 (0.02,0.05)	30	0.06 (0.04,0.08)	19	0.04 (0.03,0.07)	4	0.02 (0.01,0.06)	7	0.02 (0.01,0.04)
DQB1*03:19	15	0.02 (0.01,0.03)	42	0.05 (0.04,0.07)	4	0.01 (0.00,0.02)	2	0.00 (0.00,0.02)	11	0.06 (0.03,0.11)	37	0.11 (0.08,0.14)
DQB1*04:02	36	0.05 (0.04,0.07)	42	0.05 (0.04,0.07)	21	0.04 (0.02,0.06)	19	0.04 (0.03,0.07)	15	0.08 (0.05,0.13)	19	0.05 (0.03,0.08)
DQB1*05:01	96	0.13 (0.11,0.16)	100	0.12 (0.10,0.14)	69	0.13 (0.10,0.16)	51	0.12 (0.09,0.15)	26	0.14 (0.10,0.20)	45	0.13 (0.10,0.17)
DQB1*06:02	90	0.13 (0.10,0.15)	118	0.14 (0.12,0.17)	56	0.10 (0.08,0.13)	47	0.11 (0.08,0.14)	34	0.19 (0.13,0.25)	65	0.19 (0.15,0.23)
DQB1*06:03	33	0.05 (0.03,0.06)	37	0.04 (0.03,0.06)	28	0.05 (0.04,0.07)	28	0.06 (0.04,0.09)	5	0.03 (0.01,0.06)	8	0.02 (0.01,0.04)
DRB1*01:01	51	0.07 (0.05,0.09)	38	0.04 (0.03,0.06)	47	0.09 (0.07,0.12)	28	0.06 (0.04,0.09)	4	0.02 (0.01,0.05)	8	0.02 (0.01,0.04)
DRB1*03:01	75	0.10 (0.08,0.12)	75	0.09 (0.07,0.11)	61	0.11 (0.09,0.14)	47	0.11 (0.08,0.14)	14	0.06 (0.04,0.11)	22	0.06 (0.04,0.09)
DRB1*03:02	13	0.02 (0.01,0.03)	23	0.03 (0.02,0.04)	1	0.00 (0.00,0.01)	4	0.01 (0.00,0.02)	12	0.06 (0.03,0.10)	18	0.05 (0.03,0.08)
DRB1*04:01	53	0.07 (0.05,0.09)	40	0.05 (0.03,0.06)	48	0.09 (0.07,0.12)	27	0.06 (0.04,0.09)	5	0.02 (0.01,0.05)	11	0.03 (0.01,0.05)
DRB1*04:04	20	0.03 (0.02,0.04)	23	0.03 (0.02,0.04)	13	0.02 (0.01,0.04)	21	0.05 (0.03,0.07)	7	0.03 (0.01,0.07)	1	0.00 (0.00,0.01)
DRB1*07:01	101	0.13 (0.11,0.16)	81	0.10 (0.08,0.12)	74	0.14 (0.11,0.17)	56	0.13 (0.10,0.16)	27	0.13 (0.08,0.18)	25	0.07 (0.04,0.10)
DRB1*08:04	14	0.02 (0.01,0.03)	16	0.02 (0.01,0.03)	2	0.00 (0.00,0.01)	4	0.01 (0.00,0.02)	12	0.06 (0.03,0.10)	12	0.03 (0.02,0.06)
DRB1*09:01	15	0.02 (0.01,0.03)	16	0.02 (0.01,0.03)	4	0.01 (0.00,0.02)	5	0.01 (0.00,0.03)	11	0.05 (0.03,0.09)	10	0.03 (0.01,0.05)
DRB1*11:01	40	0.05 (0.04,0.07)	77	0.09 (0.07,0.11)	29	0.05 (0.04,0.08)	38	0.09 (0.06,0.12)	11	0.05 (0.03,0.09)	38	0.10 (0.07,0.14)
DRB1*11:02	8	0.01 (0.00,0.02)	20	0.02 (0.01,0.04)	2	0.00 (0.00,0.01)	0	0.00 (0.00,0.01)	6	0.03 (0.01,0.06)	19	0.05 (0.03,0.08)
DRB1*11:04	28	0.04 (0.02,0.05)	25	0.03 (0.02,0.04)	26	0.05 (0.03,0.07)	22	0.05 (0.03,0.08)	2	0.01 (0.00,0.03)	3	0.01 (0.00,0.02)
DRB1*12:01	23	0.03 (0.02,0.05)	15	0.02 (0.01,0.03)	10	0.02 (0.01,0.03)	4	0.01 (0.00,0.02)	13	0.06 (0.03,0.10)	11	0.03 (0.01,0.05)
DRB1*13:01	37	0.05 (0.03,0.07)	55	0.06 (0.05,0.08)	26	0.05 (0.03,0.07)	24	0.06 (0.04,0.08)	11	0.05 (0.03,0.09)	30	0.08 (0.06,0.11)
DRB1*13:02	38	0.05 (0.04,0.07)	46	0.05 (0.04,0.07)	23	0.04 (0.03,0.06)	21	0.05 (0.03,0.07)	15	0.07 (0.04,0.11)	23	0.06 (0.04,0.09)
DRB1*15:01	67	0.09 (0.07,0.11)	63	0.07 (0.06,0.09)	55	0.10 (0.08,0.13)	44	0.10 (0.07,0.13)	12	0.06 (0.03,0.10)	12	0.03 (0.02,0.06)
DRB1*15:03	26	0.03 (0.02,0.05)	47	0.06 (0.04,0.07)	2	0.00 (0.00,0.01)	3	0.01 (0.00,0.02)	24	0.11 (0.07,0.16)	45	0.12 (0.09,0.16)

**TABLE 2:** Logistic regression of GAD and Class II. Alleles filtered at greater ≥0.05; Correction factors for each gene: DPA1: 4 (*p* < 0.0125); DPB1: 7 (*p* < 0.007), DQA1: 10 (*p* < 0.005), DQB1: 10 (*p* < 0.005) and DRB1: 16 (*p* < 0.003). Bold indicates significant *p*-value.

	Full GAD		White GAD		Black GAD	
Allele	OR (95% CI)	*p*-value	OR (95% CI)	*p*-value	OR (95% CI)	*p*-value
DPA1*01:03	0.60 (0.49,0.73)	**5.92e-07**	0.82 (0.61,1.10)	0.182	0.65 (0.45,0.95)	0.0247
DPA1*02:01	1.49 (1.17,1.91)	**0.00137**	1.04 (0.74,1.48)	0.817	1.65 (1.10,2.48)	0.0158
DPA1*02:02	1.78 (1.22,2.59)	**0.00284**	2.37 (1.13,4.97)	0.0218	0.90 (0.55,1.45)	0.660
DPA1*03:01	2.05 (1.10,3.81)	0.0240	2.15 (0.58,7.89)	0.250	1.35 (0.65,2.79)	0.421
DPB1*01:01	1.21 (0.91,1.61)	0.188	0.59 (0.33,1.07)	0.0803	0.94 (0.62,1.41)	0.767
DPB1*02:01	0.87 (0.64,1.18)	0.360	1.04 (0.73,1.49)	0.822	0.83 (0.44,1.55)	0.552
DPB1*03:01	0.93 (0.65,1.32)	0.677	1.06 (0.70,1.61)	0.771	1.02 (0.46,2.28)	0.964
DPB1*04:01	0.57 (0.46,0.71)	**7.81e-07**	0.70 (0.53,0.92)	0.0101	0.54 (0.33,0.88)	0.0140
DPB1*04:02	0.97 (0.71,1.33)	0.857	1.17 (0.81,1.70)	0.395	1.02 (0.54,1.91)	0.958
DPB1*17:01	1.76 (1.00,3.11)	0.0492	1.47 (0.62,3.48)	0.378	1.51 (0.69,3.31)	0.303
DPB1*18:01	1.12 (0.48,2.58)	0.793	1.00 (.,.)		0.63 (0.27,1.51)	0.303
DQA1*01:01	0.82 (0.58,1.18)	0.285	0.87 (0.57,1.31)	0.499	1.28 (0.57,2.91)	0.551
DQA1*01:02	1.20 (0.94,1.52)	0.139	1.03 (0.74,1.43)	0.881	1.06 (0.72,1.57)	0.768
DQA1*01:03	1.04 (0.69,1.58)	0.851	0.99 (0.60,1.63)	0.956	1.54 (0.61,3.89)	0.364
DQA1*01:05	1.35 (0.74,2.49)	0.329	1.41 (0.50,3.97)	0.509	0.97 (0.43,2.17)	0.936
DQA1*02:01	0.74 (0.55,1.00)	0.0484	0.94 (0.66,1.34)	0.721	0.58 (0.32,1.06)	0.0748
DQA1*03:01	0.90 (0.60,1.34)	0.599	1.12 (0.70,1.79)	0.626	0.69 (0.29,1.62)	0.389
DQA1*03:03	1.06 (0.72,1.56)	0.759	0.86 (0.52,1.42)	0.557	1.30 (0.66,2.56)	0.454
DQA1*04:01	1.06 (0.69,1.62)	0.783	0.97 (0.51,1.85)	0.926	0.82 (0.45,1.49)	0.508
DQA1*05:01	0.86 (0.62,1.19)	0.370	0.91 (0.61,1.36)	0.653	0.85 (0.45,1.62)	0.624
DQA1*05:05	1.08 (0.82,1.43)	0.580	1.19 (0.84,1.67)	0.326	1.10 (0.65,1.85)	0.720
DQB1*02:01	0.84 (0.60,1.18)	0.316	0.91 (0.61,1.36)	0.653	0.77 (0.39,1.54)	0.464
DQB1*02:02	0.98 (0.72,1.33)	0.893	1.10 (0.73,1.64)	0.651	0.80 (0.49,1.31)	0.371
DQB1*03:01	0.94 (0.72,1.22)	0.636	1.18 (0.87,1.61)	0.287	0.92 (0.47,1.81)	0.820
DQB1*03:02	0.93 (0.65,1.33)	0.694	0.95 (0.60,1.49)	0.818	1.03 (0.54,1.97)	0.928
DQB1*03:03	0.68 (0.41,1.14)	0.143	0.77 (0.43,1.38)	0.382	0.90 (0.26,3.16)	0.871
DQB1*03:19	2.45 (1.35,4.44)	**0.00327**	0.61 (0.11,3.36)	0.569	1.84 (0.91,3.73)	0.0897
DQB1*04:02	1.01 (0.65,1.56)	0.969	1.10 (0.60,2.01)	0.754	0.67 (0.34,1.30)	0.233
DQB1*05:01	0.88 (0.65,1.20)	0.420	0.89 (0.59,1.32)	0.552	0.87 (0.51,1.49)	0.619
DQB1*06:02	1.15 (0.86,1.55)	0.336	1.03 (0.69,1.54)	0.885	0.99 (0.61,1.58)	0.952
DQB1*06:03	0.97 (0.60,1.56)	0.897	1.25 (0.72,2.15)	0.429	0.84 (0.29,2.44)	0.752
DRB1*01:01	0.66 (0.43,1.00)	0.0526	0.72 (0.45,1.16)	0.182	1.18 (0.35,4.00)	0.796
DRB1*03:01	0.88 (0.63,1.22)	0.439	0.94 (0.63,1.40)	0.754	0.91 (0.46,1.83)	0.794
DRB1*03:02	1.53 (0.79,2.96)	0.212	4.97 (0.55,44.81)	0.153	0.88 (0.43,1.81)	0.726
DRB1*04:01	0.67 (0.44,1.01)	0.0561	0.69 (0.43,1.11)	0.123	1.30 (0.44,3.85)	0.633
DRB1*04:04	1.02 (0.56,1.86)	0.954	2.08 (1.02,4.26)	0.0447	0.11 (0.01,0.86)	0.0357
DRB1*07:01	0.70 (0.52,0.95)	0.0199	0.93 (0.65,1.31)	0.665	0.50 (0.28,0.90)	0.0197
DRB1*08:04	1.01 (0.49,2.10)	0.975	2.48 (0.45,13.65)	0.298	0.55 (0.24,1.28)	0.168
DRB1*09:01	0.95 (0.47,1.90)	0.874	1.54 (0.41,5.82)	0.522	0.54 (0.23,1.26)	0.153
DRB1*11:01	1.77 (1.19,2.62)	0.00487	1.60 (0.99,2.60)	0.0571	2.28 (1.11,4.68)	0.0246
DRB1*11:02	2.17 (0.96,4.89)	0.0629	1.00 (.,.)		1.86 (0.74,4.69)	0.189
DRB1*11:04	0.78 (0.45,1.36)	0.385	1.04 (0.58,1.86)	0.899	0.87 (0.14,5.31)	0.883
DRB1*12:01	0.56 (0.29,1.09)	0.0896	0.48 (0.15,1.55)	0.221	0.46 (0.20,1.07)	0.0719
DRB1*13:01	1.33 (0.87,2.02)	0.191	1.14 (0.64,2.02)	0.654	1.59 (0.80,3.18)	0.189
DRB1*13:02	1.08 (0.69,1.69)	0.743	1.12 (0.61,2.06)	0.705	0.88 (0.44,1.77)	0.720
DRB1*15:01	0.82 (0.58,1.17)	0.282	0.98 (0.65,1.48)	0.916	0.55 (0.24,1.28)	0.168
DRB1*15:03	1.57 (0.98,2.52)	0.0607	1.85 (0.31,11.16)	0.503	1.10 (0.66,1.86)	0.708

**FIGURE 1:**
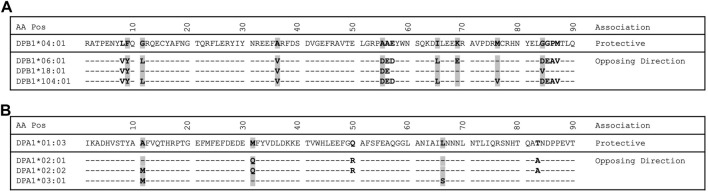
Protein alignment of select DPB1 and DPA1 alleles. **(A)** DPB1 alignment. **(B)** DPA1 alignment. Residues that are polymorphic are bold. Residues with a gray background indicate a pocket residue.

Since the A1 and B1 genes of -DP and -DQ are both polymorphic and exist in particular haplotype combinations (i.e., DQA1∼DQB1 and DPA1∼DPB1) resulting in heterodimers of α and β chains, the binding sites formed from the α and β chains of a given combination are functionally meaningful. The relative frequencies and *p*-values of the different DQA1∼DQB1 and DPA1∼DPB1 haplotypes in our control and AD populations are shown in Table 3. No remarkable differences are observed in the distribution of DQA1∼DQB1 haplotypes. A number of DPA1∼DPB1 haplotypes appear to have significant differences between the two groups (Table 3). Of note is that one of the DPA1∼DPB1 haplotypes (DPA1*01:03∼DPB1*04:01) is associated with protection and highly significant (*p* = 4.76 × 10^−07^). The same DPA1*01:03 and DPB1*04:01 alleles were found to be associated with protection with significant differences when evaluated as independent alleles (Table 2). Additionally, we noticed (Table 3; Figure 1A) that there are several DPB1 alleles (DPB1*06:01, 18:01 and 104:01) found in a haplotype with the DPA1*01:03 allele that have opposing direction (OR>1), suggesting association with AD.

**TABLE 3:** Haplotypes for the entire dataset for HLA molecules with alpha and beta chains sequenced. Correction factor for DPA1∼DPB1 is 20 (*p* < 0.0025), DQA1∼DQB1 is 24 (*p* < 0.00208). Significant *p*-values are bold.

DPA1∼DPB1 haplotype	All subjects		White		Black	
	OR (95% CI)	*p*-value	OR (95% CI)	*p*-value	OR (95% CI)	*p*-value
DPA1*01:03∼DPB1*02:01	0.84 (0.61,1.16)	0.272	1.03 (0.70,1.50)	0.887	0.84 (0.44,1.63)	0.568
DPA1*01:03∼DPB1*03:01	0.95 (0.66,1.38)	0.774	1.05 (0.68,1.64)	0.809	0.99 (0.43,2.45)	0.988
DPA1*01:03∼DPB1*04:01	0.56 (0.45,0.71)	**4.09E-07**	0.69 (0.52,0.91)	0.00706	0.55 (0.32,0.93)	0.0153
DPA1*01:03∼DPB1*04:02	0.81 (0.56,1.16)	0.225	1.15 (0.77,1.73)	0.473	0.51 (0.18,1.48)	0.155
DPA1*01:03∼DPB1*06:01	1.60 (0.54,5.31)	0.350	1.99 (0.57,7.79)	0.221		
DPA1*01:03∼DPB1*104:01	4.12 (1.14,22.41)	0.016				
DPA1*01:03∼DPB1*18:01	1.42 (0.54,3.98)	0.434			0.84 (0.32,2.40)	0.712
DPA1*02:01∼DPB1*01:01	1.09 (0.75,1.60)	0.634	0.38 (0.17,0.82)	0.00731	1.23 (0.73,2.11)	0.425
DPA1*02:01∼DPB1*05:01	0.62 (0.15,2.28)	0.411				
DPA1*02:01∼DPB1*09:01	1.04 (0.26,4.35)	0.942				
DPA1*02:01∼DPB1*10:01	0.87 (0.26,2.92)	0.794	0.88 (0.22,3.25)	0.828		
DPA1*02:01∼DPB1*11:01	1.26 (0.50,3.37)	0.590				
DPA1*02:01∼DPB1*13:01	1.47 (0.74,3.02)	0.243	1.24 (0.52,2.94)	0.587		
DPA1*02:01∼DPB1*131:01	5.75 (1.29,52.56)	0.009			3.50 (0.78,32.19)	0.0820
DPA1*02:01∼DPB1*14:01	2.47 (0.83,8.80)	0.075	2.76 (0.87,10.20)	0.0517		
DPA1*02:01∼DPB1*17:01	2.08 (1.10,4.14)	0.017	1.71 (0.62,4.96)	0.245	1.85 (0.75,5.19)	0.155
DPA1*02:02∼DPB1*01:01	1.45 (0.90,2.35)	0.108			0.83 (0.48,1.45)	0.483
DPA1*02:02∼DPB1*05:01	2.81 (1.07,8.63)	0.022	2.50 (0.77,9.40)	0.0856		
DPA1*03:01∼DPB1*04:02	2.07 (1.01,4.51)	0.033			1.71 (0.58,6.06)	0.299
DPA1*03:01∼DPB1*105:01	1.46 (0.48,4.90)	0.466				

DQA1∼DQB1 haplotype						
DQA1*01:01∼DQB1*05:01	0.84 (0.58,1.21)	0.328	0.84 (0.54,1.30)	0.423	1.43 (0.59,3.80)	0.403
DQA1*01:02∼DQB1*06:04	0.79 (0.40,1.56)	0.458	0.92 (0.39,2.09)	0.825		
DQA1*01:02∼DQB1*05:01	0.97 (0.35,2.73)	0.954			0.66 (0.23,1.95)	0.380
DQA1*01:02∼DQB1*05:02	1.12 (0.57,2.23)	0.714	0.61 (0.21,1.63)	0.281	1.76 (0.53,7.52)	0.322
DQA1*01:02∼DQB1*06:02	1.18 (0.87,1.61)	0.271	1.05 (0.68,1.62)	0.801	1.02 (0.63,1.69)	0.929
DQA1*01:02∼DQB1*06:09	1.86 (0.83,4.46)	0.103	1.86 (0.58,6.39)	0.236		
DQA1*01:03∼DQB1*06:01	1.21 (0.44,3.48)	0.685	0.76 (0.20,2.67)	0.638		
DQA1*01:03∼DQB1*06:03	0.93 (0.55,1.59)	0.778	1.10 (0.60,2.01)	0.751		
DQA1*01:04∼DQB1*05:03	0.58 (0.25,1.28)	0.141	0.57 (0.21,1.41)	0.189		
DQA1*01:05∼DQB1*05:01	0.98 (0.48,2.02)	0.951	1.44 (0.41,5.22)	0.514	0.57 (0.22,1.54)	0.207
DQA1*02:01∼DQB1*02:02	0.88 (0.61,1.28)	0.482	1.11 (0.70,1.75)	0.649	0.71 (0.36,1.40)	0.271
DQA1*02:01∼DQB1*03:03	0.48 (0.23,0.94)	0.021	0.68 (0.32,1.37)	0.248		
DQA1*03:01∼DQB1*03:02	0.87 (0.57,1.33)	0.496	1.05 (0.64,1.73)	0.834	0.64 (0.24,1.78)	0.327
DQA1*03:02∼DQB1*03:03	1.76 (0.47,8.02)	0.351				
DQA1*03:03∼DQB1*02:02	1.27 (0.50,3.39)	0.582			0.66 (0.23,1.95)	0.380
DQA1*03:03∼DQB1*03:01	0.87 (0.48,1.60)	0.636	0.83 (0.41,1.62)	0.555		
DQA1*03:03∼DQB1*03:02	0.95 (0.40,2.29)	0.897			2.17 (0.58,12.15)	0.223
DQA1*04:01∼DQB1*03:19	1.58 (0.47,6.05)	0.407				
DQA1*04:01∼DQB1*04:02	0.93 (0.55,1.57)	0.769	0.88 (0.39,1.93)	0.735	0.71 (0.33,1.57)	0.340
DQA1*05:01∼DQB1*02:01	0.86 (0.61,1.23)	0.400	0.91 (0.59,1.40)	0.655	0.87 (0.41,1.87)	0.684
DQA1*05:05∼DQB1*03:01	0.87 (0.63,1.22)	0.406	1.25 (0.85,1.82)	0.230	0.62 (0.29,1.36)	0.183
DQA1*05:05∼DQB1*03:19	2.79 (1.32,6.42)	0.004			2.15 (0.89,5.96)	0.0724
DQA1*06:01∼DQB1*03:01	5.78 (1.30,52.82)	0.009				

Adapting the same approach previously applied in a study of tuberculoid leprosy (Zerva et al., 1996), whereby alleles with opposite directionality of association can guide our search for the relevant residues that influence disease process, we identified 14 residues (8, 9, 11, 36, 55, 56, 57, 65, 69, 76, 84, 85, 86 and 87) in the β1 domain to be different between the DPB1*04:01 and the group of DPB1*06:01, 18:01 and 104:01 alleles (Figure 1A). Of those residues in the β chain, the residue at position 84 is part of pocket 1, residue at position 76 is part of pocket 2, residues 69 and 76 are part of pocket 4, residues 65 and 69 are part of pocket 7, residues 9, 36 and 55 are part of pocket 9 and residue 11 is part of pocket 6 (Table 4). In a similar fashion we identified the pocket residues that are different between the DPA1 alleles associated with disease and protection (Figure 1B). The DPA1*01:03 allele was associated with protection and the other 3 alleles, DPA1*02:01, *02:02 and *03:01, were associated with disease (Table 2). For the DPα chain we find that residues 11 and 66 are part of pocket 6, while residue 31 is part of pocket 1 (Table 4). Thereafter, and knowing the different residues of the pockets associated with AD or protection, we evaluated the distribution of these pocket residues in the whole population of control and AD subjects. In the analysis of pocket residue combinations shown in Table 5 we found that amino acid combinations of pocket residues of the DP dimer may be associated with protection or AD. In terms of AD association or protection, it appears that while there are pockets influenced by both DP α and β chain residues, like pocket 1 (α31, β84) and 6 (α11, β11), there are others that are influenced only by residues of the β chain, like pocket 4 (β69 and 76), pocket 7 (β65 and 69) and pocket 9 (β36 and 55). More specifically the combinations of α31M + (β84G or β84V) at pocket 1, α11A+ β11G at pocket 6, β36A+ β55A at pocket 9, and β69K + β76M at pocket 4, are associated with lower risk and therefore protective, while the combination of α31Q + β84D is associated with increased risk of disease (Table 5). The performed analysis was targeted and involved predetermined positions and amino acids generated from the described analytical approach, hence, the only correction factor here is the number of different estimates, that is 10, and shown in Table 5.

**TABLE 4:** Pocket Residues for DP proteins. Residues that are bold are identified to be polymorphic in Figure 1.

Pocket	Alpha chain residues	Beta chain residues
1	24, **31**, 32, 43, 52, 53, 54, 55	80, 83, **84**
2	9	76, 79, 80
3	22, 54, 58	
4	9, 62	13, 24, 26, 68, **69**, 72, **76**
5		69
6	**11**, 62, 65, **66**	**11**, 13, 26, 28
7	65, 69	26, 28, 45, 59, **65**, 68, **69**
8	65, 68, 69	
9	69, 72, 73, 76	9, 28, 35, **36**, **55**, 59

**TABLE 5:** HLA-DP Pocket Residue Analysis. Correction factor of 10 (*p* < 0.005; bold text).

Chain	Pocket	Residues	All		White		Black	
			OR (95% CI)	*p*-value	OR (95% CI)	*p*-value	OR (95% CI)	*p*-value
DPA1+DPB1	P1	α31M + (β84G or β84V)	0.57 (0.46,0.70)	**1.87E-07**	0.79 (0.59,1.04)	0.094	0.53 (0.35,0.78)	**1.65E-03**
DPA1+DPB1	P1	α31Q + β84D	1.63 (1.31,2.04)	**1.11E-05**	1.21 (0.88,1.66)	0.237	1.54 (1.05,2.28)	0.027
DPA1+DPB1	P6	α11A+ β11G	0.54 (0.44,0.68)	**3.25E-08**	0.64 (0.48,0.85)	**2.08E-03**	0.64 (0.44,0.92)	0.018
DPA1+DPB1	P6	α11M + β11L	1.54 (0.56,5.19)	0.430	2.49 (0.24,53.9)	0.457	1.03 (0.33,4.10)	0.957
DPB1	P9	β36A+ β55A	0.72 (0.58,0.88)	**0.002**	0.68 (0.52,0.88)	0.004	0.67 (0.45,0.98)	0.039
DPB1	P9	Β36V + β55D	1.22 (0.99,1.51)	0.057	1.28 (0.99,1.67)	0.065	1.43 (0.98,2.12)	0.064
DPB1	P7	β65I + β69K	0.75 (0.61,0.93)	0.008	0.74 (0.57,0.96)	0.027	0.68 (0.46,1.00)	0.054
DPB1	P7	β65L + β69E	1.77 (0.68,5.12)	0.260	1.76 (0.55,6.02)	0.340	2.13 (0.31,42.04)	0.501
DPB1	P4	β69K + β76M	0.65 (0.53,0.80)	**5.00E-05**	0.79 (0.61,1.03)	0.085	0.62 (0.41,0.92)	0.017
DPB1	P4	β69E + (β76V or β76I)	1.23 (0.80,1.92)	0.356	1.11 (0.65,1.90)	0.695	1.57 (0.61,4.81)	0.382

Regarding the analysis of the DRB1 gene, we focused on the polymorphic pocket residues of DRβ chain. The DRB1 polymorphisms specific to pocket residues are shown in Supplemental Table 2, none of these differences were statistically significant after *p*-value correction (*n* = 63, threshold *p*-value = 0.0008). Also considering that the DQA1∼DQB1 haplotyping did not reveal any significant associations, we evaluated the polymorphic pocket residues of the DQA1 and DQB1 genes independently (Supplemental Table 2). Accounting for the 25 DQA1 and 45 DQB1 comparisons, none of these residues demonstrated a statistically significant difference after correction.

Assessing polymorphisms in the non-exon 2 sequences of the DRB1, DQA1, and DQB1 alleles revealed no significant differences (Supplemental Table 2). However, DPα and β chains, did reveal other additional residues being of significance located in exon 3 and other segments of the molecule like transmembrane and cytoplasmic domains (Supplemental Table 2). These residues were the following: DPα111, 127, 160 and 228 and DPβ96 and 170. To assess whether any of these polymorphisms outside of the α1 and β1 domains were conferring independent risk from those identified within the α1 and β1 domains in the previous analysis we performed an assessment of LD. The amino acids in these positions of the non-α1 and β1 domains were found to be in variable LD with polymorphisms of the α1 and β1 domains (Supplemental Table 3). Since very strong LD is demonstrated between the non-α1 and β1 residues with at least one of the α1 or β1 residues it is unclear as to whether they confer an independent contribution to susceptibility or protection.

Additionally, the frequency of SNP rs9277534 polymorphisms, that denote relative expression of the DPB1 gene, were evaluated. The G polymorphism for rs9277534 SNP located in the 3’ UTR of the DPB1 gene, reflecting higher expression, was associated with AD (OR = 1.45, *p* = 4.71e-04), while the alternative, A, reflecting lower expression, was associated with protection (OR = 0.69, *p* = 4.71e-04) (Table 6). It was also noted that neither G nor A was associated with any of the two racial groups included in our study.

**TABLE 6:** Logistic regression analysis of DPB1 expression SNP rs9277534. Bold text indicates significant *p*-value.

SNP	Full dataset		White		Black	
	OR (95% CI)	*p*-value	OR (95% CI)	*p*-value	OR (95% CI)	*p*-value
rs9277534 A (Low)	0.69 (0.56,0.85)	**4.71E-04**	0.90 (0.68,1.19)	0.444	0.75 (0.51,1.10)	0.140
rs9277534 G (High)	1.45 (1.18,1.78)	**4.71E-04**	1.12 (0.84,1.48)	0.444	1.33 (0.91,1.95)	0.140

Considering the strong linkage disequilibrium of these A/G polymorphisms with DPB1 alleles, the LD of the A/G SNP polymorphism with each of the positions from Table 5 and Supplemental Table 2 with at least one significant association, was assessed (Table 7) (Schöne et al., 2018). The underlying principle here is that, while DPB1 alleles may be in LD with the 3’ UTR SNP rs9277534 polymorphism, that does not mean that individual residues of the DP molecule will necessarily be in LD with either G or A of the rs9277534 SNP. A particular residue may be present on several alleles, some of which may be in LD with the G and some others with A SNP. Indeed, it was observed that while some of these residues are in LD with the A (DPβ84G and β76M) or G polymorphism (DPβ84D), not all residue polymorphisms of the binding pockets, with significance for either susceptibility or protection, are in LD with the rs9277534 SNP (DPβ36A, β55A, β69K) (Table 7). Upon assessing the LD between exon 3 polymorphisms and the rs9277534 SNP, it was found that there is very strong LD between the two (DPβ96, β170) (Table 7), suggesting interrelated functionalities between the expression of DPβ molecule and the β2 domain itself.

**TABLE 7:** Relationship between DPB1 positions with at least one residue of significance for protection/association of AD (Table 5; Supplemental Table 2) with DPB1 alleles found in this study and the 3’ UTR SNP rs9277534. Alleles with frequency >0.05 are bold and underlined. (p) = protection; (s) = susceptibility.

Position	Residue	rs9277534 ‘A’		rs9277534 ‘G’	
		Alleles	*R* ^2^	Alleles	*R* ^2^
β11	G (p)	**02:01**, 02:02, **04:01**, **04:02**, 23:01, 39:01, 40:01, 49:01, 106:01, 126:01, 138:01, 535:01	0.257	**01:01**, 05:01, 15:01, 16:01, **18:01,** 19:01, 90:01, 100:01, 350:01, 417:01	0.257
	L	**17:01**, 30:01, 55:01, 133:01, 907:01	0.257	**03:01**, 06:01**, 09:01**, 10:01, 11:01, 13:01, 14:01, 20:01, 21:01, 29:01, 35:01, 36:01, 45:01, 85:01, 104:01, 131:01, 519:01	0.257
β36	A (p)	**04:01**, 39:01, 40:01, 49:01, 126:01, 133:01, 907:01	0.000	**01:01**, 11:01, 13:01, 15:01, 85:01, 90:01, 350:01, 417:01, 519:01	0.000
	V	**02:01**, 02:02, **04:02**, **17:01**, 23:01, 30:01, 55:01, 106:01, 138:01, 535:01	0.000	**03:01**, 05:01, 06:01, **09:01**, 10:01, 14:01, 16:01, **18:01**, 19:01, 20:01, 21:01, 29:01, 35:01, 36:01, 45:01, 100:01, 104:01, 131:01	0.000
β55	A (p)	**04:01**, 23:01, 39:01, 40:01, 55:01, 126:01, 133:01, 138:01, 907:01	0.000	**01:01**, 11:01, 13:01, 15:01, 85:01, 90:01, 350:01, 417:01, 519:01	0.000
	D	**02:01**, **04:02**, **17:01**, 49:01	0.003	**03:01**, 06:01, **09:01**, 10:01, 14:01, 16:01, **18:01**, 20:01, 29:01, 35:01, 45:01, 104:01, 131:01	0.003
	E	02:02, 30:01, 106:01, 535:01	0.024	05:01, 19:01, 21:01, 36:01, 100:01	0.024
β69	E	**02:01**, 02:02, **17:01**, 30:01, 55:01, 106:01, 133:01, 535:01	0.014	06:01, **09:01**, 10:01, 13:01, 16:01, 19:01, 21:01, 29:01, 131:01, 519:01	0.014
	K (p)	**04:01**, **04:02**, 23:01, 39:01, 40:01, 49:01, 126:01, 138:01	0.004	**01:01**, **03:01**, 05:01, 14:01, **18:01**, 20:01, 35:01, 36:01, 45:01, 85:01, 90:01, 100:01, 104:01, 350:01, 417:01	0.004
	R	907:01	0.030	11:01, 15:01	0.030
β76	I	106:01, 133:01, 535:01	0.044	13:01, 19:01, 519:01	0.044
	M (p)	**02:01**, 02:02, **04:01**, **04:02**, **17:01**, 23:01, 30:01, 39:01, 40:01, 49:01, 55:01, 126:01, 138:01, 907:01	0.639	05:01, 06:01, 11:01, 15:01, 16:01, **18:01**, 20:01, 21:01, 36:01, 85:01, 100:01, 131:01, 350:01	0.639
	V	*<No alleles>*	0.549	**01:01**, **03:01**, **09:01**, 10:01, 14:01, 29:01, 35:01, 45:01, 90:01, 104:01, 417:01	0.549
β84	G (p)	**02:01,** 02:02**, 04:01, 04:02,** 23:01, 39:01, 49:01, 126:01, 138:01	0.835	100:01, 350:01	0.835
	V (p)	40:01	0.028	15:01, **18:01**	0.028
	D (s)	**17:01**, 30:01, 55:01, 106:01, 133:01, 535:01, 907:01	0.753	**01:01**, **03:01**, 05:01, 06:01**, 09:01**, 10:01, 11:01, 13:01, 14:01, 16:01, 19:01, 20:01, 21:01, 29:01, 35:01, 36:01, 45:01, 85:01, 90:01, 104:01, 131:01, 417:01, 519:01	0.753
β96	K (s)	*<no alleles>*	1.0	**01:01**, **03:01**, 05:01, 06:01**, 09:01**, 10:01, 11:01, 13:01, 14:01, 15:01, 16:01, **18:01**,19:01, 20:01, 21:01, 29:01, 35:01, 36:01, 45:01, 85:01, 90:01, 100:01, 104:01, 131:01, 350:01, 417:01, 519:01	1.0
	R (p)	**02:01,** 02:02**, 04:01, 04:02, 17:01**, 23:01, 30:01, 39:01, 40:01, 49:01, 55:01, 106:01, 126:01, 133:01, 138:01, 535:01, 907:01	1.0	*<no alleles>*	1.0
β170	I (s)	*<no alleles>*	1.0	**01:01**, **03:01**, 05:01, 06:01**, 09:01**, 10:01, 11:01, 13:01, 14:01, 15:01, 16:01, **18:01**, 19:01, 20:01, 21:01, 29:01, 35:01, 36:01, 45:01, 85:01, 90:01, 100:01, 104:01, 131:01, 350:01, 417:01, 519:01	1.0
	T (p)	**02:01,** 02:02**, 04:01, 04:02, 17:01**, 23:01, 30:01, 39:01, 40:01, 49:01, 55:01, 106:01, 126:01, 133:01, 138:01, 535:01, 907:01	1.0	*<no alleles>*	1.0

Regarding the analysis of our data using the TCE groups for the DP alleles we found moderate correlation between TCE group 3 and multiple pocket residues to be associated with protection from AD: P1 (α31M + (β84G or β84V); r = 0.53), P4 (β69K + β76M; r = 0.44), P6 (α11A + β55G; r = 0.59) and P9 (β 36A + β84V; r = 0.43), while we also found moderate correlation between TCE group 1 and P1 residues associated with AD (α31Q + β84D; r = 0.40). Table 8 shows the relative association of each one of the TCE groups for protection or susceptibility to AD, whereby TCE group 3 is associated with protection from AD (OR = 0.64, *p* = 0.00100) and TCE group 1 is associated with susceptibility to AD (OR 1.76, *p* = 0.0097).

**TABLE 8:** Associations between DPB1 TCE groups and Atopic Dermatitis in the GAD cohort. Correction factor of 3 (*p* < 0.0167; bold text).

DPB1 TCE group	Full dataset		White		Black	
	OR (95% CI)	*p*-value	OR (95% CI)	*p*-value	OR (95% CI)	*p*-value
TCE Group 1	1.76 (1.16,2.74)	**0.00969**	1.05 (0.56,1.97)	0.869	2.03 (1.07,4.18)	0.0401
TCE Group 2	1.34 (0.99,1.82)	0.0571	1.40 (0.98,2.01)	0.0655	1.86 (0.97,3.83)	0.0734
TCE Group 3	0.64 (0.49,0.83)	**0.00100**	0.74 (0.53,1.03)	0.0729	0.49 (0.29,0.79)	**0.00467**

In PEER, a cohort of children with AD used as a replication cohort, we confirmed that children with DPA1*01:03 and DPB1*04:01, and the DPA1*01:03∼DPB1*04:01 haplotype, were less likely to have AD as compared to the GAD controls. For this comparison we used controls from the GAD cohort because the PEER cohort was comprised of only AD cases which were used to study the progression of disease over time. As in many genetic association studies, such as GWAS, data from a standard set of controls are often used for different case comparisons because the underlying premise is that the “public” control group is representative of the underlying relevant non-diseased population (Mitchell et al., 2014). We also confirmed the susceptibility role of P1: α31Q + β84D and protective role of P1: α31M + (β84G or β84V) and P6: α11A + β11G. However, the DP β chain residues of pockets 4 and 9 were not confirmed. (Table 9). Furthermore, it was confirmed that the distribution of the SNP rs9277534 polymorphisms A/G in the PEER cohort was such that the A remains to be associated with protection and ‘G’ with susceptibility. None of the associations between TCE groups and AD were replicated in the PEER cohort. No correction was applied to the *p* values, as this study was a replication confirming the findings of the GAD cohort.

**TABLE 9:** Replication of GAD case findings using PEER subjects for the significant associations found in the GAD cohort. Bold indicates significant *p*-value.

			All subjects		White		Black	
Alleles			OR (95% CI)	p-value	OR (95% CI)	p-value	OR (95% CI)	p-value
DPA1*01:03			0.74 (0.61,0.91)	**0.00421**	1.05 0.78,1.42)	0.767	0.60 (0.41,0.87)	**0.00833**
DPB1*04:01			0.75 (0.61,0.93)	**0.00941**	0.95 (0.73,1.22)	0.673	0.53 (0.31,0.89)	**0.0167**
DQB1*03:19			2.59 (1.50,4.80)	**0.00124**	0.54 (0.07,2.81)	0.483	2.45 (1.29,5.11)	**0.0103**
DPA1∼DPB1 haplotype								
DPA1*01:03∼DPB1*04:01			0.72 (0.58,0.91)	**0.00439**	0.93 (0.71,1.20)	0.555	0.52 (0.30,0.91)	**0.0134**
Pockets								
Chain	Pocket	Residues						
DPA1+DPB1	P1	α31M + (β84G or β84V)	0.72 (0.59,0.90)	**0.00291**	0.85 (0.65,1.10)	0.223	0.68 (0.46,1.01)	0.0636
DPA1+DPB1	P1	α31Q + β84D	1.29 (1.03,1.61)	**0.0245**	0.95 (0.69,1.31)	0.765	1.42 (0.98,2.09)	0.0678
DPA1+DPB1	P6	α11A+ β11G	0.66 (0.53,0.82)	**0.000173**	0.76 (0.58,1.00)	0.0540	0.60 (0.40,0.88)	**0.00954**
DPB1	P9	β36A+ β55A	0.94 (0.76,1.16)	0.571	0.95 (0.74,1.22)	0.707	0.85 (0.58,1.24)	0.393
DPB1	P4	β69K + β76M	0.82 (0.67,1.01)	0.0647	0.92 (0.72,1.19)	0.540	0.85 (0.57,1.26)	0.417
DPB1 Expression SNP								
rs9277534_A_Low			0.78 (0.63,0.96)	**0.0176**	0.87 (0.67,1.13)	0.296	0.85 (0.58,1.24)	0.399
rs9277534_G_High			1.25 (1.02,1.54)	**0.0338**	1.12 (0.86,1.46)	0.401	1.14 (0.78,1.67)	0.493
DPB1 TCE Groups								
TCE Group 1			1.11 (0.70,1.77)	0.658	0.76 (0.38,1.47)	0.431	1.39 (0.71,2.87)	0.351
TCE Group 3			0.95 (0.72,1.25)	0.705	0.96 (0.69,1.33)	0.788	0.86 (0.49,1.45)	0.569

## 4 Discussion

HLA Class II genes are involved in the formation of molecules integral to the presentation of antigens to CD_4_ T cells and as a result are part of immune mediated responses and illnesses. We conducted a case-control study of individuals with and without AD using high-resolution NGS sequencing of genes in the HLA Class II region. We also evaluated individuals by White or Black race. After statistical adjustment for multiple comparisons, we found no associations between HLA-DRB1, DQA1 or DQB1 alleles or any of their positional residues or amino acids with AD. Analysis of DPA1 and DPB1 alleles, DPA1/DPB1 dimers and pocket residues had significantly different distributions between the control and disease cases. This evaluation allows for assessing the role of positional residues and of specific amino acids in a way that is independent of specific -DP alleles. This evaluation is meaningful because individual alleles may not be significantly associated with AD, while specific positional amino acids that are shared among multiple alleles can demonstrate significant associations between control and disease cases. Considering that the operational entities influencing peptide binding are the pocket residues, this type of analysis allows for a better assessment of the sub-molecular components of an HLA molecule that contributes to disease or protection. The findings were replicated in a second group of AD cases from the PEER cohort for the P1 and P6 pockets and their respective residues. Replication did not confirm the involvement of the P4 and P9 pocket residues. More specifically, P1; α31Q + β84D are associated with a susceptibility role and P1: α31M + (β84G or β84V) and P6: α11A + β11G are associated with a protective role. In summary, -DPA1 and B1 genomic variation is associated with AD but no other Class II genes are associated with AD.

Accounting for the relative expression of the DPs on the cell surface, whereby the residue β84G, which was associated with protection, is in LD with the rs9277534 3’ UTR SNP polymorphism indicating lower expression (A), while the residue (β84D) associated with disease is in LD with the SNP reflecting higher expression (G), it becomes apparent that a combination of polymorphisms in the coding region, together with cell surface expression of the DP molecule and the presented non-self antigen, may set the stage for the ensuing immune response that results in protection or disease.

It should also be mentioned that the absolute LD identified between exon 3 – transmembrane region-3 UTR SNP rs9277534 (residue 96R-170T-SNP A or residue 96K-170I-SNP G) of DPB1 in our population (Table 7), denotes a possible interdependence between these polymorphisms and expression as reflected by SNP rs9277534 in the 3’ UTR of the DPB1 gene. It is unclear as to how these non-exon 2 polymorphisms may coordinate functionalities critical for the molecule, but this LD character contrasts to the somehow strong but not absolute LD between the rs9277534 SNP and exon 2 polymorphisms that comprise the part of the DP that serves as a receptor for peptides and T cell receptor interactions (Table 7). Furthermore, residue DPβ11G that is associated with protection is in rather weak LD with exon 3 polymorphisms (β96 and β170, Table 8) and also the rs9277534 SNP A (Table 7). It therefore appears that association with protection may or may not depend entirely on the low levels of expression of the DP molecules. If we assume that protection can be an active state of the immune response, like susceptibility is, then there may be T cell responses of regulatory nature that actively diminish the immune response; these mechanisms may be influenced not only by structural components but also expression patterns and the peptides presented by the DP molecule. Lower expression of these DP structural components can be compatible with the protective role; nevertheless, the details of the interactions between structure and expression are not entirely clear. It should be clarified that in our study we claim associations of either pocket residues or SNPs denoting expression with susceptibility or protection but we do not demonstrate that these residues or SNP polymorphisms are engaged directly in specific mechanisms resulting in disease susceptibility or protection.

In conclusion, the LD observed among polymorphisms in exonic sequences (particularly exon 3 and to lesser extent exon 2) and non-coding regions (intron 2 or 3’ UTR) is an indicator suggesting coordinated functionalities maintained through the evolutionary history of the DPB1, connected through the different segments of the DPB1 genomic sequences. A hypothesis, therefore, can be developed that those particular structural components of DPs, along with their increased or reduced expression, form the basis for the pathophysiology of AD. It remains, that because of the LD between the residues and expression components, it is unclear what the exact mechanistic role each may play and what their exact interactions may be. It should be noted that the HLA-DP loci are not in LD with the other HLA Class I loci or HLA Class II loci, as there are at least four recombination hotspots located between the HLA-DP locus and the next closest HLA locus studied in this project, HLA-DQB1 (Jeffreys et al., 2001; Cullen et al., 2002; Miretti et al., 2005). The DPB1 alleles, amino acid residues and the expression marker are not correlated with the HLA Class I alleles or residues (Margolis et al., 2021b) that were either protective of or associated with AD from this same dataset (data not shown). Therefore, the involvement/engagement of the Class I and the Class II associated or protective elements in AD pathophysiology are rather independent and do not reflect any linkage disequilibrium effect.

In our study, the HLA typing was done by NGS. Unlike previous studies of AD, we identified and fully characterized both the A and the B genes of DQ and DP loci, allowing a better identification of pocket residues. This combined evaluation is important because polymorphisms on both A and B genes contribute to the binding site and therefore influence peptide binding. In the past, in HLA and disease association studies the characterization of the DQA1 and DPA1 genes was not performed routinely, either because it was not feasible at the time or because there was a belief that their polymorphism was limited and therefore inconsequential. Since the majority of HLA and disease associated studies lack this DPA1, DQA1 gene characterization, our understanding and contribution of the possible role of α pocket residues is unclear for the different HLA and disease association studies performed thus far. In our study, the β84D is in combination with α31Q (Table 5) and therefore it is likely that, this combined structural features of the DP molecule, along with its high expression may contribute to disease susceptibility. Recent studies of Type 1 diabetes mellitus provide another example of the importance of characterizing both A and B gene polymorphisms which show associations with both A and B genes of DQ and DP loci (Erlich et al., 2008; Noble, 2015; Enczmann et al., 2021).

The DP pockets and residues associated, in our study, with AD P1β84 and P6β11, have been previously identified by Castelli *et al* as influencing the binding of synthetic peptides originating from allergens, viral and tumor antigens to DP molecules (Castelli et al., 2002). The P1 and P6 pockets accommodate the main anchor residues of foreign peptides interacting with DPs. Even though the Castelli *et al* study (Castelli et al., 2002) does not address the contributions of the α chain residues, considering that these pockets are composed of polymorphic α chain residues as well, it is not unlikely that whatever the effect of the pockets, is a resultant of influences originating from both chains. It therefore becomes likely that a combination of DP α and β pocket polymorphisms, as they interact with different antigens, form the complex that initiates T cell responses. Indeed, in our study we find that not only β chain residues are associated with protection or disease, but also α chain residues (α11A, α31Q, and α31M).

Furthermore, the different HLA-DPB1 alleles are organized into T cell epitope (TCE) functional groups (Zino et al., 2007; Crivello et al., 2015). Each one of these TCE groups of DPB1 alleles is characterized by distinct structural features that differentiate one from the other group and influence the peptides bound to the DP protein. Most recently, the immunopeptidome of the alleles that belong to each one of these groups has been characterized (van Balen et al., 2020; Meurer et al., 2021; Laghmouchi et al., 2022). Considering that in our study of AD we have identified HLA-DPA1 and DPB1 alleles and specific residues relevant to the disease, we were interested to investigate as to whether any of the structural elements (alleles or residues) of the DP molecules correlated with the TCE groups, therefore, establishing a relationship between the DP structural elements of our study with the functional grouping of DPs including the peptide repertoire that characterizes each one of the TCE groups. Indeed, we found in the GAD cohort that those particular alleles belonging to a specific TCE group, having common structural features and a unique immunopeptidome bound to these alleles, correlate with the structural elements we found through our independent approach in the same cohort. However, due to lack of reproducibility with the PEER group we do not expand on this observation and refrain from a final conclusion. Nevertheless, it is very likely that the structural elements, identified as being relevant with the disease, correlate to particular DP TCE groups. As such, the immunopeptidome characterizing the different TCE groups may be instructive in identifying peptides involved with atopic dermatitis; the exact structural details within a particular TCE group that may play a role in atopic dermatitis remain unclear.

Atopic illnesses, like atopic dermatitis, are associated with T_H_2 dominant cellular response and often IgE antibody production in response to the presence and presentation of specific allergens. The activation of the T_H_2 results in the production of IL-4 and IL-13 cytokines. These cytokines are associated with the acute phase of AD and can also result in epidermal barrier dysfunction (Gong et al., 2020). The physiologic effect of these cytokines are diminished by IL-4 blocking agents that are currently being used to treat moderate to severe AD (Beck et al., 2014). The strength of the immune response to an antigen is likely related to genetic and environmental factors as well as the type and intensity of allergen.

Allergen presentation is influenced by HLA Class II and, in this study, we demonstrate that HLA Class II genetic variation at the level of allele and receptor epitope is associated with both an increased risk and decreased risk of AD. We do not currently know which allergens bind and with what intensity to the epitopes effected by the described genetic variation. We did previously show in an *in silico* study that HLA Class II epitope variation could result in differential binding of auto allergens suspected of being associated with AD (Gong et al., 2020). However, when an allergen is suspected to be associated with AD, it should now be possible to determine how it might interact with the epitopes described in this study and more precisely determine if it is likely to be associated with AD. Furthermore, agents that influence -DP binding might have an impact on AD.

As with all epidemiologic studies, this study has limitations. Although this study is the largest study of its kind to use NGS to genotype HLA Class II genes and then evaluate their associations with AD, we were under-powered to evaluate less common HLA alleles. For this reason, we *a priori* limited our analyses to alleles with a frequency of ≥0.05. We believe that this strategy is important for understanding the effect of HLA on the population at large. However, it is possible that important information about how HLA Class II allelic variation affects immune response with respect to AD might be gleaned by evaluating less frequent alleles. To evaluate these alleles, large cohorts will be needed that are designed to focus on less common alleles. Study subjects were classified by self-described race and not by genetic ancestry. It is possible that genetic admixture is not fully accounted for by self-described race and could have added bias to our results. However, it is important to note that we have previously shown that in an evaluation of a similar population, self-described race was highly concordant with an assessment of genetic ancestry (Margolis et al., 2012; Abuabara et al., 2020; Biagini et al., 2022). In addition, many of our findings had similar effect estimates in both races. The origin of the GAD is primarily from academic dermatology offices and as such patients were more likely to have treatment resistant AD than patients seen in general practice. As a result, it is possible that our results may not generalize to all clinical sites and to individuals with less treatment resistant AD. Our genotyping did not phase the A/B genes of the DQ or DP loci, so we used a well-known algorithm, haplo.stat to form our DPA1∼DPB1 and DQA1∼DQB1 haplotypes. It is possible that there was some inaccuracy in the estimation of haplotypes. However, as a sensitivity analysis, we used an alternative approach for coding the haplotypes that does not rely on the EM algorithm and found very similar results (see Supplemental Table 4). Finally, it is possible that our findings are not primarily associated with AD but other co-morbid illnesses that are part of the atopic March like asthma and seasonal allergies (Zino et al., 2007). However, it is generally believed that AD occurs before these illnesses (Kapoor et al., 2008).

In summary, we conducted a case-control study of individuals with AD and controls using NGS for the characterization of the three classic HLA Class II genes. NGS allows for a more thorough interrogation of Class II genes and found that the DP molecule is relevant to AD and that specific pockets and residues within these pockets, along with their expression levels, play a critical role in both susceptibility and protection.

## Data Availability

The HLA Class II genotypes for the GAD and PEER cohorts presented in the study are deposited in the Zenodo repository, https://zenodo.org/record/7565999 (DOI: 10.5281/zenodo.7565999).
